# Revolutionizing Tissue Engineering through Mirroring Cell Niche and Application of Natural Compounds

**Published:** 2017-05

**Authors:** Hosein Shahsavarani, Mohammad Ali Shokrgozar

**Affiliations:** National Cell Bank of Iran, Pasteur Institute of Iran, Tehran, Iran

Considering the high prevalence of severe organ failures due to the cancer, congenital anomaly, or trauma, and the consequent needs for tissue transplantation, deficiencies in tissues and organs are a huge challenge for regenerative medicine at the moment. More than 40 years have passed since the term ‘tissue engineering’ was created as a new therapeutic means, which may overcome the drawbacks involved in the current artificial tissue or organ transplantation. Nevertheless, application of regenerated tissues is still restricted mainly owing to the cost, poor biocompatibility, low bio-functionality, as well as immune rejection. Researchers have come a long way to make safer neotissues from cells with the support of new biomaterials, recombinant proteins, and the lower dose of growth factors for medical research or even clinical trials, but some critical problems should still be resolved for the use in human patients. Thus, these issues have lead to the emergence of a new concept that focuses on looking for an alternative approach to reconstruct tissue and organ using natural, safer and cost effective methods.

A major challenge in tissue engineering and cell culture is the use of serum, animal (Xeno) products, and recombinant proteins in the media or extracellular matrix, which are rather expensive, ethically questioned or problematic for researchers to study the mechanisms of a specific biological cascade. Attempts to imitate physiology of the human organs in the laboratory are getting closer to capturing their intricacies, which is needed for clinical applications. Therefore, to try to tackle the aforementioned problems, it is desirable to exploit natural compounds instead of synthetic materials as an alternative way to assemble functional constructs that restore, maintain, or improve damaged tissues or even whole organs.

Natural products, including plant derivatives and marine compounds, have widely demonstrated their worth as a cost-effective source of molecules and functional bio-composites with therapeutic potentials over thousands of years. In recent years, rapid advances in nanotechnology in addition to the extraction of the newly discovered natural small molecules opened an arena to produce functional human organs in sufficient structure and size at low cost for clinical applications.

## Physical differentiation of stem cells with imprinting method

**Fig. 1:**
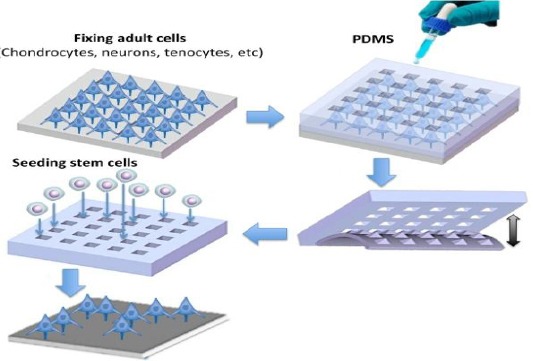
Cell-imprinted substrates direct fate of stem cells

In the human body, morphological structures and mechanical loadings direct the cell fate during embryonic developments. Considering significant effects of physical interactions on differentiation of stem cells into mature cells, a new paradigm has been proposed for mirroring regeneration of various kinds of tissues, including chondrocytes, neurons, tenocytes, and semi-fibroblasts via analyzing matrix production and controlling mechanical properties of cell-scaffold construct. Cell imprinting technique has found to recapitulate the physiological niche of the cells, in the hopes that they will enable cells to foster actual tissue or organ development processes. In this method, smart nano-environment platforms has been made by cell-imprinted substrates based on mature and dedifferentiated cells as templates and demonstrated their potential for differentiation, re-differentiation, and trans-differentiation. Scaffolds that have been fabricated using imprinting technique are in actual structure dimensions of the target tissue and can better mimic the *in vivo* cellular microenvironment, which benefits the localization, attachment, proliferation, and differentiation of stem cells. In this method, we actually mirror the dynamic environment of the cells in a specific tissue, and scaffolds for cell cultures are made using similar manufacturing techniques to those for sculpture (Fig. 1). Production of a rubbery 3D structure close to the target cell niche, which mimics physical stimuli for differentiation, guides the cells to pass the correct path of development. This method would be an effective and promising way to regulate any cell phenotype *in vitro* with remarkable potential applications in tissue engineering and cell-based therapies.

## Herbal products: an alternative source for regenerative medicine

Animal-derived materials have the risk of inducing hypersensitivity reactions and pathogenic contamination, whereas the risk associated with the use of human-derived proteins includes disease transmission. The production of recombinant human proteins using plant material is required, thus providing a feasible alternative without the risk of disease transmission or variability concerns. Large-scale production of growth factors in plants, as a new alternative to other production process, addresses limitations in terms of scale-up, cost-efficiency, and the purity of these proteins.

Advances in extraction technology, separation science (chromatographic techniques), and analytical and spectroscopic instrumentation have extended the contribution and usefulness of plant-derived chemical or biologically active constituents in tissue engineering, as affordable resources for controlling cell fate or even plant-based extra cellular matrix. The positive effects of various herbal products on the fate of the proliferation and differentiation of human stem cell have been confirmed. These products have minimum side effects though their mechanisms of action still remain unknown. Moreover, the large-scale production of recombinant human collagen type I has been reported in tobacco, which has made a homogenic, heterotrimeric, thermally stable, functional scaffold for tissue engineering.

**Fig. 2:**
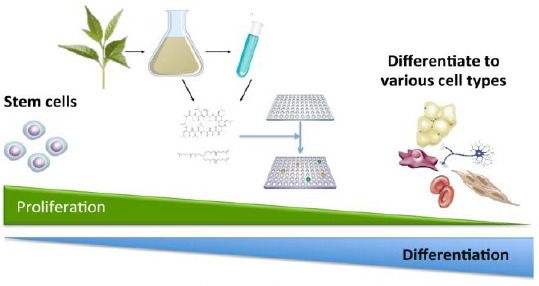
Directing cell fate using plant-derived chemical

In near future, biologically active plant-derived chemical compounds can be expected to play an increasingly significant role in the commercial development of culture systems and in the new products for regulating cell proliferation, growth or even directing cell fate (Fig. 2). Both plant-derived active molecules and scaffold materials, which promote proper maturation and differentiation of stem cells, may provide a novel source of raw material for tissue engineering with low risk of allergic response and disease transmission. Investigation on the effects of plant-derived compounds on differentiation, maintenance, and regeneration of stem cells will have to be pursued *in vitro*, as well as in preclinical and clinical settings.

## Use of marine biomaterials in tissue engineering

In spite of progress in making artificial scaffolds for tissue engineering, providing a sufficient supply of oxygen for the cells still remains a challenge. As a solution, exploiting photosynthetic organisms to fulfill metabolic oxygen requirements of the cells has recently been proposed to contribute to the success of biomaterial-based tissue culture. For instance, co-culturing the cells with single-cell photosynthetic algae, *Chlamydomonas* *reinhardtii*, which can be found in water, could address the problem of hypoxia, by exposure to the light. Therefore, it is an unlimited biocompatible source of oxygen that can be easily regulated according to the tissue needs by modulating the intensity of light applied.

*In vivo* mouse experiments have indicated no immune-based rejection with rather high survival rates; however, it would be desirable if human/plant chimeric tissue was formed and grafted to human body to address the problems associated with the lack of vascularization and oxygen and/or nutrient deprivations. Further, soft porous biomaterials such as collagen are unable to endure fixation with sutures, which may be due to their low tearing strength. Marine bio-adhesive protein compounds, which can strongly attach to different surfaces, even under wet conditions, have recently attracted much attention as a cost-effective source for surface modification to improve cell culture efficiency in tissue engineering.

Combining all the aforementioned strategies would modulate mechanical, chemical and electrical cues or even stiffness of the target tissue for better reproduction of the developmental signals that cells receive in body. Indeed, mimicking cell niches through cell imprinting and the use of organic products deliver what regenerative medicine aspires; allowing decisive experiments to understand the mechanisms and the potentials to cure the diseases in need of tissue or organ transplantation.
